# A Web-Based Serious Game on Delirium as an Educational Intervention for Medical Students: Randomized Controlled Trial

**DOI:** 10.2196/games.9886

**Published:** 2018-10-26

**Authors:** Kiki R Buijs-Spanjers, Harianne HM Hegge, Carolien J Jansen, Evert Hoogendoorn, Sophia E de Rooij

**Affiliations:** 1 Department of Geriatric Medicine University Medical Center Groningen University of Groningen Groningen Netherlands; 2 IJsfontein Amsterdam Netherlands

**Keywords:** delirium, education, medical students, serious game

## Abstract

**Background:**

Adequate delirium recognition and management are important to reduce the incidence and severity of delirium. To improve delirium recognition and management, training of medical staff and students is needed.

**Objective:**

In this study, we aimed to gain insight into whether the serious game, Delirium Experience, is suited as an educational intervention.

**Methods:**

We conducted a three-arm randomized controlled trial. We enrolled 156 students in the third year of their Bachelor of Medical Sciences degree at the University Medical Centre Groningen. The Game group of this study played Delirium Experience. The Control D group watched a video with explanations on delirium and a patient’s experience of delirious episodes. The Control A group watched a video on healthy aging. To investigate students’ skills, we used a video of a delirious patient for which students had to give care recommendations and complete the Delirium Observations Screening Scale and Delirium Rating Scale R-98. Furthermore, students completed the Delirium Attitude Scale, the Learning Motivation and Engagement Questionnaire, and self-reported knowledge on delirium.

**Results:**

In total, 156 students participated in this study (Game group, n=51; Control D group, n=51; Control A group, n=55). The Game group scored higher with a median (interquartile range) of 6 (4-8) for given recommendations and learning motivation and engagement compared with the Control D (1, 1-4) and A (0, 0-3) groups (*P*<.001). Furthermore, the Game group scored higher (7, 6-8) on self-reported knowledge compared with the Control A group (6, 5-6; *P*<.001). We did not find differences between the groups regarding delirium screening (*P*=.07) and rating (*P*=.45) skills or attitude toward delirious patients (*P*=.55).

**Conclusions:**

The serious game, Delirium Experience, is suitable as an educational intervention to teach delirium care to medical students and has added value in addition to a lecture.

## Introduction

Delirium is an acute neuropsychiatric syndrome often experienced by older hospitalized patients. It is characterized by altered attention, awareness, and cognition. Delirium has serious consequences such as increased length of hospital stay, functional decline, institutionalization, and mortality [1]. Adequate delirium recognition and management are important to reduce the incidence and severity of delirium [2,3]. To improve delirium recognition and management, training of medical staff and students is needed [4] as timely recognition is crucial [2]. Lack of delirium awareness, knowledge, and education were the most commonly reported barriers to improving the recognition of delirium (risk) and the hospital care for delirious patients [5]. Current educational interventions focus merely on increasing knowledge and skills in recognition of delirium but do not seem to be effective enough [6,7]. It was suggested that educational interventions on delirium should have a broader scope to target (1) the attitude of the medical staff and students toward delirious patients; (2) the understanding of patients’ needs; and (3) the translation of this knowledge into the practice of offering good health care to delirious patients [7,8]. Future educational interventions on delirium should not only have a broader scope addressing these 3 objectives but also focus on teaching methods with students actively involved and supportive technologies with sufficient feedback loops [6,7].

Serious games may be an opportunity to meet this demand for new educational interventions. Serious games are games developed and intended to provide playful learning experiences, which can be transferable to or applicable in real-life settings [9]. Serious games are often more effective compared with regular health care educational interventions [10] or assessments [11]. However, there is a lack of effect studies [12] and assessment [13] of good quality on serious games.

Delirium Experience is a recently designed serious game that uses video simulation [14], which is intended to train and educate medical students on how to take better care of delirious patients. As both serious games [15] and simulation-based learning [16,17] provide learning spaces in which learners can safely practice, Delirium Experience might serve as a new educational intervention by addressing the need for a focus on caregiver attitude and the application of knowledge to the care of delirious patients.

In this study, we aimed to gain insight into whether Delirium Experience is suited as an educational intervention for medical students regarding skills in advising care for delirious patients, skills in screening and rating of delirium symptoms, and improving the attitude toward delirious patients. Additionally, we aimed to gain insight into the possible effects of Delirium Experience on learning motivation and engagement, as well as self-reported knowledge on delirium.

## Methods

### Design and Study Population

We conducted a three-arm randomized controlled trial. The study population consisted of undergraduate medical students at the University Medical Centre Groningen (UMCG). To be included in this study, participants had to (1) be in their third year of preclinical education in December 2016; (2) sign up for the practical on delirium; and (3) sign the informed consent form. The UMCG offers an undergraduate program of 6 years—3 years of preclinical and 3 years of clinical education. Preclinical medical students at the UMCG select 1 of 4 different learning communities with different, in-depth focus during their medical education (global health, sustainable care, intramural care, and molecular medicine). At the moment, the UMCG third-year preclinical medical curriculum on delirium is based on lectures and literature. However, educators of the UMCG emphasize the need for a more practice-based education before students enter their clinical education.

Students started with the conventional lecture on delirium. Thereafter, students could voluntarily sign up for the practical on delirium, in which the study conditions took place. The practicals were given in three separate classrooms of the University of Groningen. Each study condition had a separate classroom. All students had the opportunity to join the practical on delirium, including students who did not wish to participate in the study. Students were informed about the study in the description of the practical. This practical description explained that the practical was divided over 3 different groups for research purposes but did not explain the different study conditions. Students were not aware that the serious game, Delirium Experience, was one of the study conditions, in order not to influence the motivation to sign up for the practical. All students were provided a license of Delirium Experience after the practical so they could play the serious game. Data were collected and analyzed anonymously.

We used SPSS 23.0 (IBM Inc) for stratified block randomization (block size of 6) to allocate participants into one of the three research groups [18]. Learning communities represented the 4 different strata used. All participants who signed up for the practical were randomly allocated to one of the groups. They subsequently received an email indicating the classroom in which they were expected. As our research subjects consisted of medical students who could voluntarily sign up for both the practical and the study, registration of the trial was not necessary in accordance with the ICMJE (International Committee of Medical Journal Editors) recommendations. Multimedia Appendix 1 shows the CONSORT-EHEALTH checklist.

### Intervention and Control Groups

We designed three different practicals on delirium, which represented the study conditions. Only the intervention group, the Game group, played Delirium Experience [14]. Delirium Experience is a serious game focusing on delirium both from a patient’s and a caregiver’s perspective (watch the trailer in Multimedia Appendix 2). The goal of Delirium Experience is to allow players to learn how to take better care of delirious patients. The game tries to achieve this by giving players insight into what a patient experiences during delirious episodes and how your actions as a caregiver influence the experience of the patient. Delirium Experience was based on the delirium guidelines used in the United Kingdom [19] and the Netherlands [20] and on stories of patients who suffered from delirious episodes. The game was developed with personnel who were specialists in developing serious games, designing education, and treating delirium, all working closely together. Usability was tested by a group of care professionals during the development. Based on their suggestions and feedback, the final version of the game was made. Completing the game once takes approximately 20 minutes; in these 20 minutes, one experiences 4 days as a caregiver and the corresponding 4 nights as the patient. During the daytime, as a caregiver, the player has to take care of a delirious patient and can choose different actions. Depending on the actions one chooses, the delirious episodes of the patient differ in severity, and one gets different actions to choose from the next day. Hence, if one performs poorly as a caregiver, the severity of delirious episodes increases, and the next day, one has fewer actions to choose from compared with a caregiver who performed well. Players who perform poorly have their actions limited to only the most important actions to decrease the level of difficulty. Furthermore, players receive feedback every other day in the game on how they performed and how they could improve as a caregiver before they switch to the patient’s perspective.

We compared this Game group with two other groups, one with and one without information about delirium. The first control group, Control D group, watched a video on delirium, which explained delirium causes, symptoms, diagnosis, treatment, and pathology. Contrary to the serious game, the video did not ask for active involvement of students; thus, students were not able to try different scenarios. Furthermore, this group watched a second video of a patient’s experience explaining his suffering from delirious episodes.

The second control group, Control A group, watched a more general video on healthy aging. This video did not have any specific information on delirium and how to take care of delirious patients; each session took 20 minutes.

### Outcome Measurements

At baseline, before the intervention started, all participants completed a form including questions on sex, age, experience with older and delirious patients, learning community, self-reported knowledge on delirium, *Which mark (0-10) would you give your knowledge on delirium?*, and attendance at the lecture. Primary and secondary outcome measures were assessed directly after the intervention or control condition.

The primary outcome of this research was assessment of the skills acquired by students in advising care for delirious patients, in which students describe how they would manage delirium in practice. In this outcome, students could show their understanding of patients’ needs and be able to translate this knowledge into practice [7,8]. To measure skills in advising care, all participants observed an interview of a delirious patient and were asked to give 3 written recommendations for the care of this patient. A predefined rubric-form was used to assess all given recommendations as rubric-forms can enhance the reliability of assessors’ scoring [21]. The rubric-form was based on the Dutch delirium guidelines [20]. Recommendations were assessed independently by two researchers, and a weighted kappa was calculated. To ensure blinding of the assessors, data on intervention and control groups were removed from the assessed recommendations. Each recommendation could receive 0 (incorrect or not mentioned), 1 (topic mentioned), 2 (nonspecific recommendation), or 3 (specific recommendation) points from the 10 different domains of the Dutch delirium guidelines (range, 0-9 points) [20].

Subsequently, several secondary outcomes were measured. First, use of screening and rating instruments for delirium was measured. Participants completed the Delirium Observations Screening Scale (DOSS) [22] and Delirium Rating Scale R-98 (DRS-R-98) [23,24] for the patient in the observed interview. Both scales are widely accepted and applied tests for the recognition and severity assessment of delirium. Second, attitude toward delirious patients was measured using the Delirium Attitude Scale. The Delirium Attitude Scale is based on the Dementia Attitude Scale [25]. Items regarding creativeness, enjoyment of life, and coping skills were replaced by items focusing on the experiences of delirium. This resulted in a 19-item 7-point Likert scale (range, 19-133 points). *I feel confident around people with delirium* and *I would avoid an agitated person with delirium* are examples of statements used in the Delirium Attitude Scale. Third, learning motivation and engagement were measured using the Motivation and Engagement Questionnaire to evaluate learning experiences [26], a 9-item 5-point Likert scale (range, 9-45 points). Examples of statements used in this questionnaire are as follows: *It was challenging to perform well in this practical* and *I liked this way of learning.* Finally, participants were asked to self-report their knowledge on delirium (range, 0-10 points).

### Statistical Methods

We checked data for normality by judging histograms, skewness, and kurtosis. We analyzed discrete variables using chi-square test. Furthermore, continuous variables were analyzed using one-way analysis of variance (ANOVA) in case of normal distribution and Kruskal-Wallis in case of a nonnormal distribution. *P*<.05 was considered statistically significant for the results of the chi-square and one-way ANOVA or Kruskal-Wallis tests. In case of significant results regarding outcome measurements, specific post hoc or Mann-Whitney *U* tests were performed to investigate differences between the (1) Game group and Control D group or (2) Game group and Control A group. Furthermore, a Bonferroni correction for two tests was used for the Mann-Whitney *U* test; therefore, *P*<.025 was considered statistically significant for the results of the Mann-Whitney *U* test.

## Results

**Figure 1:**
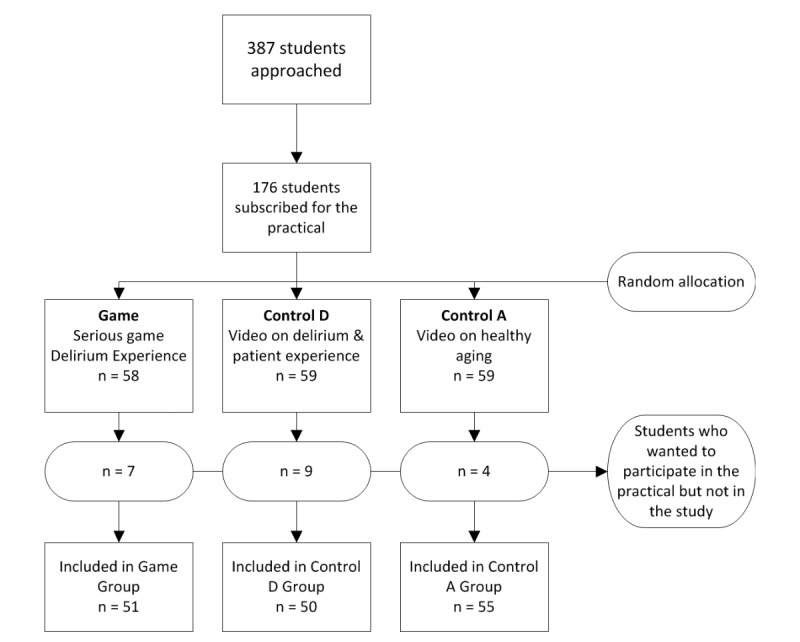
Flowchart of approached students and participants.

In total, 176 of 387 students subscribed for the practical on delirium in December 2016. Of these 176 students, 156 signed the informed consent form and participated in the study (Figure 1). The 20 students who declined to sign the informed consent form still participated in the practical but were not included in the study. Students did not have to give a reason why they declined to sign the informed consent form. We compared playing a serious game (Game group) to either watching a video on delirium in combination with a video of a patient’s experience (Control D group) or watching a video on healthy aging (Control A group). Data on students’ characteristics and outcome measures were not normally distributed. The median age (interquartile range [IQR] 25-75) of all participants was 20 (20-21) years, and 75% (117/156) participants were females. No differences were found between the research groups regarding baseline variables, as presented in Table 1.

**Table 1:** Baseline variables.

Characteristics		Total participants (N=156)	Game^a^ (n=51)	Control D^a^ (n=50)	Control A^a^ (n=55)
Age in years^b^, median (IQR^c^)		20 (20-21)	21 (20-21)	20 (20-21)	21 (20-21)
Female^d^, n (%)		117 (75.0)	37 (72.5)	40 (80.0)	40 (72.7)
Experience older patients^d^, n (%)		118 (75.6)	36 (70.6)	38 (76.0)	44 (80.0)
Experience delirious patients^d^, n (%)		48 (30.8)	17 (33.3)	15 (30.0)	17 (30.9)
**Learning community^d^, n (%)**					
	Global health	45 (28.8)	19 (37.3)	15 (30.0)	12 (21.8)
	Molecular medicine	39 (25.0)	11 (21.6)	14 (28.0)	14 (25.5)
	Sustainable care	31 (19.9)	11 (21.6)	7 (14.0)	11 (20.0)
	Intramural care	40 (25.6)	10 (19.6)	14 (28.0)	18 (32.7)
Attended lecture, n (%)		129 (82.7)	44 (86.0)	43 (86.0)	43 (78.2)
Self-reported knowledge on delirium (0-10)^b^, median (IQR)		5 (4-6)	5 (4-6)	5 (4-6)	5 (4-6)

^a^Game: Delirium Experience; Control D: video on delirium with a patient experience video; Control A: video on healthy aging.

^b^Data compared using Kruskal-Wallis test, *P*>.05.

^c^IQR: interquartile range.

^d^Data compared using chi-square test, *P*>.05.

**Table 2:** Kruskal-Wallis and Mann-Whitney *U* tests for primary and secondary outcomes for the Game (n=51), Control D (n=50), and Control A (n=55) groups.

Outcome	Game^a^	Control D^a^	Control A^a^	*P* value^b^	*P* value (G-D)^c^	*P* value (G-A)^d^
Recommendations	6 (4-8)	1 (1-4)	0 (0-3)	<.001	<.001	<.001
DOSS^e^	10 (9-11)	9 (8-10)	9 (8-11)	.07	N/A^f^	N/A
DRS-R-98^g^	14 (12-16)	13 (12-15)	14 (11-16)	.45	N/A	N/A
Attitude	92 (88-96)	94 (90-100)	92 (85-96)	.55	N/A	N/A
Learning motivation^h^	36 (32-38)	27 (24-30)	20 (15-25)	<.001	<.001	<.001
Delirium knowledge^i^	7 (6-8)	6 (6-7)	6 (5-6)	<.001	.03	<.001

^a^Data are presented as median (interquartile range 25-75); Game: Delirium Experience; Control D: video on delirium with patient experience video; Control A: video on healthy aging.

^b^Kruskall-Wallis test to compare the three groups.

^c^Mann-Whitney *U* test to compare the Game group and the Video Delirium group (*P*<.025 considered statistically significant).

^d^Mann-Whitney *U* test to compare the Game group and the Video Aging group (*P*<.025 considered statistically significant).

^e^DOSS: Delirium Observation Screening Score.

^f^N/A: not applicable.

^g^DRS-R-98: Delirium Rating Scale R-98.

^h^Learning motivation and engagement.

^i^Self-reported knowledge on delirium.

The primary outcome of this study, skills in advising care for delirious patients, was measured on the basis of the given care recommendations. The independently assessed recommendations, which were scored using the rubric-form, had a weighted kappa of .835. Disagreements were resolved through discussion.

Kruskal-Wallis tests showed differences between the three groups regarding given recommendations, *H(2)*=54.5*, P* *<*.001, learning motivation and engagement, *H(2)*=91.5, *P*<.001, and self-reported knowledge, *H(2)*=26.0, *P*<.001, as presented in Table 2. No differences were found regarding delirium screening, *H(2)*=5.2, *P*=.07, and rating *H(2)*=1.6, *P*=.45, scores or in attitude toward delirious patients, *H(2)*=5.8, *P*=.55.

Furthermore, Mann-Whitney *U* test to compare the Game group and the Control D group showed differences regarding given recommendations (*U*=466.0, *z=−* 5.58, *P*<.001) and learning motivation and engagement (*U*=302.5, *z=−* 6.61, *P*<.001) but not for self-reported knowledge (*U*=967.5, *z*=−2.18, *P*=.03). The comparison of the Game group with the Control A group showed differences in given recommendations (*U*=363.0, *z*=−6.77, *P*<.001), learning motivation and engagement (*U*=110.5, *z*=−8.18, *P*<.001), and self-reported knowledge (*U*=651.0, *z*=−4.91, *P*<.001). Participants in the Game group scored a median score (IQR 25-75) of 6 (4-8) on recommendations, whereas the Control D group had a median score of 1 (1-4), and Control A group had a median score of 0 (0-3). With regard to learning motivation and engagement, participants in the Game group had a median score of 36 (32-38) compared with a median score of 27 (24-30) for the Control D group and 20 (15-25) for the Control A group. The median mark on self-reported knowledge of the Game group was a 7 (6-8) compared with a 6 (5-6) for the Control A group.

## Discussion

### Principal Findings

In this study, we investigated the effects of a serious game, Delirium Experience, as a new educational intervention. We compared playing a serious game with watching a video with delirium explanation in combination with a patient experience video or a video on healthy aging. The results showed that the serious game had a positive effect on students’ skills in advising care for delirious patients, learning motivation and engagement, and self-reported knowledge on delirium. However, the serious game did not influence skills in screening and rating the severity of delirium. In addition, it did not affect the attitude toward delirious patients.

Although students in the group playing the serious game and the group watching the video on delirium got more familiar with the behavior of delirious patients, they were not explicitly trained in the use of the DOSS and DRS-R-98 or recognizing the delirious behavior. Furthermore, the design of Delirium Experience allows players to practice caring for a delirious patient and manage delirium instead of recognizing it. The DOSS and DRS-R-98 are used and applied in a real-life setting over 24 hours by trained and experienced health care professionals [22,24]. This might explain why we did not find differences in delirium screening and rating scores, as the medical students did not get training and patient information of 24 hours, nor did they have clinical experience with delirious patients. All participants received high scores on their attitude toward delirious patients, which could have been caused by a ceiling effect or by students answering in a socially desired way. Furthermore, attitudes can be influenced by intense emotions [27]. As participants played the game as well as they could, they probably did not see the more severe delirium scenarios, as these scenarios are only shown when the game players perform poorly.

### Implications

Conventional simulation-based educational interventions have proven to be effective but are costly owing to a large number of teachers, role-play actors, and time and space required [17]. By using Delirium Experience as a simulation-based educational intervention, students could play without the need of teachers and role-play actors. Development of serious games is costly. Delirium Experience was developed by an unrestricted grant of NutsOhra, and development costs were covered and, therefore, not relevant for educational institutes as they only pay for a license to use the game. Delirium Experience provides students with a safe environment to practice and apply attained knowledge and can be used as an educational intervention on delirium to improve skills in advising care for delirious patients. As simulation-based assessment seems to predict the clinical performance [28], this safe simulation environment might prepare preclinical students in advance for their clinical education. In addition, this study supports earlier research on the importance of including objectively obtained measurements instead of self-reported measures [12,29]; we did not find differences in the self-reported knowledge between playing the serious game and watching the delirium video, but we did find differences between these groups regarding skills in advising care for delirious patients.

### Limitations

This study has a number of limitations. First, skills in advising care for delirious patients were measured using a video of a delirious patient and using written answers instead of a real clinical situation, which would involve both the responsibility of caring for a delirious patient and the demonstration of the correct skills. However, simulation-based assessment seems to be a suitable tool for predicting clinical performances [30]. Second, there might have been selection bias in the recruitment of students, as more highly motivated students were more likely to sign up for the practical. However, due to the design of this randomized study, this could not have influenced the differences between the research groups. Furthermore, there is a slightly skewed number of students that declined to sign the informed consent form in the different groups. We cannot explain this, because students did not have to explain why they declined, nor do we have any information on these students because they were never included in the study. However, as there was only a small percentage of students that declined to sign the informed consent form, we do not expect this to influence the results. Finally, we did not perform a sample size calculation beforehand. We approached all third-year medical students. If we had not found statistically significant results due to a too low sample size or power, the study would have been extended in 2017. However, as we found significant results, the power was sufficient.

### Further Research

Further research should be performed as to whether it is possible to improve attitudes toward delirious patients with Delirium Experience. If the change in attitude can be established by more emotional and intense patient scenarios [27], Delirium Experience might improve attitudes when students are allowed to play Delirium Experience several times, including “dark play.” In a dark play situation, players show behavior in the game that in a normal care situation would be problematic [31] and increases the intensity of the delirium. In Delirium Experience, this results in adverse events and scenarios with an extremely frightened patient. Showing immoral behavior, such as dark play, in video games has already been proven to lead to improved awareness of moral norms [32]. Subsequently, it would be interesting to investigate the effect of dark play on learning outcomes such as advising care for delirious patients. Because Delirium Experience increases learning motivation, it would be interesting to investigate whether students might be more motivated to use Delirium Experience as self-study material [28] and whether the increased motivation also influences learning outcomes. Furthermore, player characteristics might influence the effectiveness and use of games and should be taken into account in future studies [33]. In addition, future studies should take into account other health care professionals and trainees to generalize the results and use of interdisciplinary games, such as Delirium Experience, and investigate whether Delirium Experience can improve timely recognition of delirium. Finally, it is important to look at long-term effects of playing a serious game and ascertain our interest in whether it can influence the strain of care in experienced health care professionals working with delirious patients.

### Conclusions

Playing Delirium Experience increases medical students’ skills in advising care for delirious patients, learning motivation and engagement, and self-reported knowledge on delirium. However, in this study, we could not show an effect on improving delirium screening and severity rating skills or on attitudes toward delirious patients after playing Delirium Experience. The serious game, Delirium Experience, is suitable as an educational intervention to teach delirium care to medical students and has added value in addition to that of a lecture.

## Appendix

Multimedia Appendix 1 CONSORT‐EHEALTH checklist (V 1.6.1).

Multimedia Appendix 2 Trailer of Delirium Experience.

## Abbreviations

ANOVA: analysis of variance
DOSS: Delirium Observations Screening Scale
DRS-R-98: Delirium Rating Scale R-98
ICMJE: International Committee of Medical Journal Editors
IQR: interquartile range
N/A: not applicable
UMCG: University Medical Centre Groningen
